# Emerging investigator series: spatially and temporally resolved lightning NO_*x*_ production rates obtained with space-based lightning detectors

**DOI:** 10.1039/d6em00482b

**Published:** 2026-09-22

**Authors:** Rebekah P. Horner, Eloise A. Marais, Lee T. Murray

**Affiliations:** a Department of Geography, University College London London UK e.marais@ucl.ac.uk; b Department of Earth and Environmental Sciences, University of Rochester Rochester NY USA

## Abstract

Lightning is a dominant free tropospheric source of nitrogen oxides (NO_*x*_), impacting climate by affecting global tropospheric ozone (O_3_) and other key oxidants. We calculate temporally and spatially resolved lightning NO_*x*_ (LNO_*x*_) production rates using lightning flash energies from space-based Lightning Imaging Sensors (LIS). Our production rates average ∼309 moles per flash (mol fl^−1^) over oceans, peaking at midday, and ∼172 mol fl^−1^ over land, peaking in the early morning. Replacing static values in the original GEOS-Chem model inventory with our spatially and hourly varying production rates yields global annual LNO_*x*_ emissions of 4.4 Tg nitrogen (N) for 2015–2019; almost 2 Tg N less than the original inventory. Annual terrestrial LNO_*x*_ emissions still exceed those over the ocean, due to more frequent lightning over land, but the relative proportion of maritime emissions increases. The largest decline in LNO_*x*_ emissions is north of 35°N in summer, as static 500 mol fl^−1^ is replaced with 150–250 mol fl^−1^. Global decline in LNO_*x*_ emissions decreases abundances of free tropospheric NO_*x*_, O_3_, the hydroxyl radical (OH) and peroxyacetyl nitrate (PAN). Hourly variability in LIS flash radiances is broadly consistent with high-frequency Geostationary Lightning Mapper (GLM) flash energies, though the comparison is limited to tropical America. Introducing hourly variability in LNO_*x*_ production rates leads to a greater than 10% localised decline in equatorial lower tropospheric O_3_. Independent validation is critically needed, given our updated LNO_*x*_ emissions offer a viable solution to model overestimates in O_3_ and OH and underestimates in the lifetime of the greenhouse gas methane.

## Abstract 2

More energetic lightning over oceans than land causes greater production of nitrogen oxides (NO_*x*_) per flash over oceans; a feature that is absent in atmospheric chemistry models.

## Graphical abstract

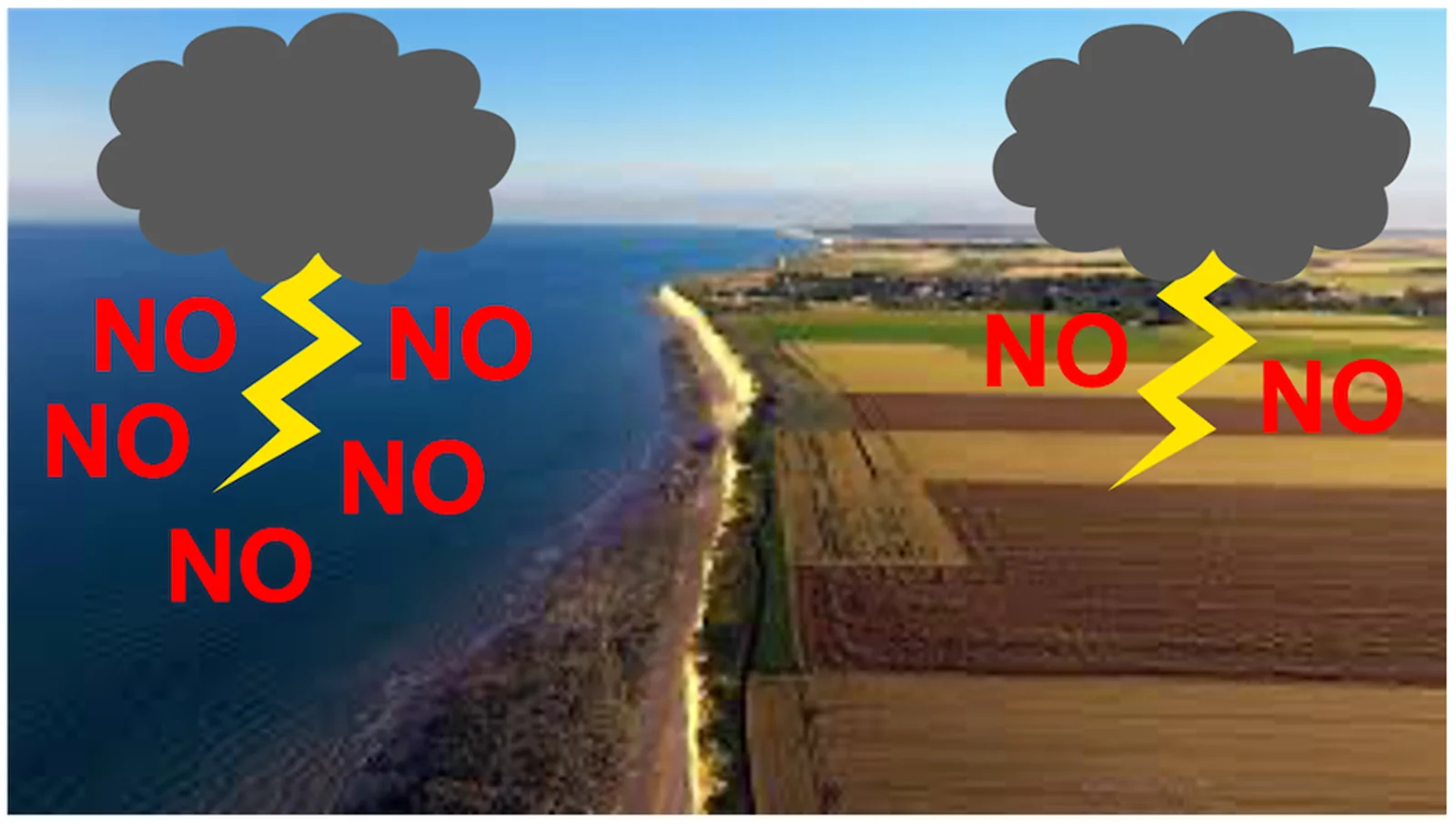

**Environmental significance**

Lightning is the dominant free tropospheric source of nitrogen oxides (NO_*x*_), affecting abundances of the greenhouse gas tropospheric ozone and the hydroxyl radical (OH), the main tropospheric oxidant that determines the lifetime of the potent greenhouse gas methane. Despite the importance of lightning, models simply use fixed values for the production of NO_*x*_ from lightning. We derive temporally and spatially varying global NO_*x*_ production rates using flash energies from space-based lightning sensors. Production rates over oceans are almost double those over land and are on a global average less than values currently used in models. Our updated rates decrease tropospheric ozone and OH, offering a solution to a long-standing overestimate of these in climate and tropospheric ozone assessment models.

## 1 Introduction

Flashes of lightning form nitrogen oxides (NO_*x*_ ≡ NO + NO_2_) from extreme heating (up to 30,000 K) of the atmosphere, causing stable N_2_ to thermally dissociate and react with already dissociated oxygen (O) atoms.^1,2^ On a global scale, lightning produces less NO_*x*_ than human activities like fossil fuel combustion, but it has a disproportionately large impact on tropospheric ozone (O_3_) and climate. Lightning is the dominant source of NO_*x*_ to the free troposphere where O_3_ production is very sensitive to NO_*x*_.^3–5^ Estimates of annual NO_*x*_ emissions from lightning cover a wide range, 2–8 Tg nitrogen (N), according to the initial extensive review by Schumann and Huntrieser.^6^ This is wider than more recent studies that establish a 3–7 Tg N range from reviewing multiple models^7^ and synthesizing the state-of-knowledge.^8^

Chemical transport models (CTMs) calculate the timing and location of lightning flashes using parameterisations that rely on meteorological variables that influence lightning occurrence, such as convective available potential energy (CAPE), cloud-top height, wind shear, convective updraft mass flux, precipitation flux, maximum vertical velocity, updraft volume, or upward ice flux.^9–13^ Most models use cloud-top height parameterisations, as this meteorological variable is more readily available than variables needed for the other parameterisations. Still, cloud-top heights are poor predictors of lightning flashes.^13^ CTMs can address issues with predicting the timing and location of lightning flashes by temporally and spatially scaling modelled flashes with climatological flashes from satellite instruments like the Optical Transient Detector (OTD) and the Lightning Imaging Sensor (LIS).^12^ Such a correction is exclusive to the GEOS-Chem CTM. There are no direct measurements of global lightning NO_*x*_ (LNO_*x*_) production rates. Almost all models rely on static values of NO_*x*_ production per flash (fl^−1^) rates, even though in reality these should vary in time and space.^14^ Models using static values include widely used global and regional CTMs such as GEOS-Chem, the Global Modeling Initiative (GMI), Weather Research and Forecasting (WRF)-Chem, the Cambridge parallelised-Tropospheric Offline Model of Chemistry and Transport (p-TOMCAT), and the UK Chemistry and Aerosols (UKCA) Model.^15–19^ An exception is the parameterized inverse relationship between lightning flash frequency and LNO_*x*_ production rates in the European Centre for Medium-Range Weather Forecasts (ECMWF)-Hamburg (ECHAM)/Modular Earth Submodel System (MESSy version 2.55) Atmospheric Chemistry (EMAC) model.^20^ Use of static LNO_*x*_ production rates introduces substantial uncertainty in regional and global LNO_*x*_ estimates and limits the accuracy of modelled past, present, and projected atmospheric composition.^21,22^

Space-based instruments such as the LIS and the Geostationary Lightning Mapper (GLM) measure the optical characteristics of lightning, in particular lightning flash energy that can be linked directly to LNO_*x*_ production.^23–28^ An LIS instrument was first deployed on NASA's low-Earth orbiting (LEO) Tropical Rainfall Measuring Mission (TRMM) and another later installed on the International Space Station (ISS). The spectral energy densities measured by the LIS in a narrow near-infrared (NIR) spectral range are used to derive flash radiances that serve as proxies for flash energy.^29,30^ The GLM, onboard NOAA's Geostationary Operational Environmental Satellite (GOES) series satellites located over the Americas, directly measures lightning flash optical energy.^26^ Recent studies have used these lightning energy data to estimate LNO_*x*_ production rates.^31,32^ Both studies used LIS and GLM flash energy data to quantify LNO_*x*_ production rates and simulate the impact of LNO_*x*_ emissions on O_3_, but coverage was limited to the continental United States (US) and neither determined sub-daily LNO_*x*_ production rates.^31,32^

Here we use lightning radiant energies from the TRMM and ISS LIS instruments to derive spatially and hourly resolved tropical and subtropical LNO_*x*_ production rates for ready inclusion in CTMs. We implement these in GEOS-Chem to test the impact on global, regional and seasonal LNO_*x*_ emissions and abundances of atmospheric oxidants, tropospheric O_3_, NO_*x*_ and the NO_*x*_ reservoir compound peroxyacetyl nitrate (PAN) that redistributes NO_*x*_ far from the emission source.

## 2 Methods

### 2.1 Space-based lightning flash and energy sensors

The TRMM LIS (TRMM-LIS) provided lightning flash and radiance data from 1998 to 2015. The instrument orbital inclination of 35° achieved latitudinal coverage of 35°N to 35°S. The LIS on the ISS (ISS-LIS) collected data from 2017 to 2023. Its orbital inclination is greater than TRMM-LIS at 51.6°, resulting in more extensive coverage of 48°N to 48°S. Characteristics consistent across both LIS instruments include a nadir spatial resolution of ∼4 km, a field of view of ∼600 km × 600 km, and a sampling frequency of 2 milliseconds. TRMM-LIS achieved full global coverage every 1–2 days and ISS-LIS every 3 days. Both instruments followed a non-sun-synchronous orbit with variable equator crossing time, enabling investigation of hourly variability in flash radiances.

We use both TRMM-LIS and ISS-LIS to take advantage of the long TRMM data record and the greater latitudinal sampling extent of ISS-LIS. To calculate 24-hour and hourly mean gridded lightning flash radiances, we use LIS radiance data from TRMM version 4.1 for 2002–2014 (https://search.earthdata.nasa.gov/, last accessed 6 August 2023) and ISS version 2.1 for 2017–2022 (https://search.earthdata.nasa.gov/, last accessed 2 January 2024). TRMM data prior to 2002 are excluded to avoid introducing systematic errors from orbital boost of the TRMM satellite in 2001.^33^ Global radiances for each local solar time (LST) hour are determined by averaging the radiance data onto a fixed 0.5° latitude × 0.625° longitude grid covering 48°N to 48°S after screening for data with a fatal quality flag (instrument, platform, processing or external error) for both the TRMM and ISS. A grid of 0.5° × 0.625° is selected to balance LIS data coverage with sufficiently resolved spatial detail. Averaging yields systematically larger values than medians,^30^ but the approach to derive LNO_*x*_ production rates (Section 2.2) includes scaling that dampens for such biases.

Since the LIS instrument detection efficiency is <100%, both TRMM and ISS flash radiances are scaled by the hourly detection efficiencies determined by Boccippio *et al.*^34^ that are summarised in Cecil *et al.*^35^ LIS efficiencies range from 70% at 12:00 LST to 90% at 01:00 LST, reflecting weaker detection during daylight hours. The same hourly scaling factors are used throughout the year, as detection efficiencies are reported to have little seasonal variability.^33,36^ We fill spatial data gaps for each hour using nearest neighbour spatial interpolation. Interpolation fills 10% of all gridsquare-hours. Most (86%) of these filled gridboxes are over the oceans at 35–48°N/S where only the shorter (7 years) ISS record is available.

We evaluate hourly variability in LIS radiant energies against hourly variability in flash energies from the GOES-16 GLM.^26^ The GLM has been mapping lightning activity consistently over this region since 2017. It has a spatial resolution of 8 km at nadir and 14 km at the edges of the field of view.^37^ The GLM provides continuous temporal coverage compared to the snapshots offered by the LIS. GLM collects data every ∼2 ms and aggregates flashes every ∼20 s.^38,39^ The continuous sampling of the GLM results in better overall detection efficiencies than the LIS.^40,41^ Given this, no scaling is applied to the GLM to correct for hourly variability in detection efficiencies. Like the LIS, we calculate hourly average GLM flash energies on a fixed 0.5° × 0.625° grid for data from 2018 to 2024 with a quality flag of 0 (categorised as “good data”).

Since the GLM directly detects lightning flash energies (in J) and the LIS detects spectral energy densities (in J sr^−1^ m^−2^ µm^−1^), we compare normalised GLM and LIS hourly energies relative to 24-hour mean energies averaged over 2019–2024 for the GLM, 2002–2014 for the TRMM and 2017–2023 for the ISS. Normalisation also accounts for an overall weaker GLM detection efficiency resulting from its much higher geostationary orbit than the low-Earth orbiting LIS instruments.^42^

The comparison is limited to Central America (40–130°W, 5–35°N) and the Gulf of Mexico (10–100°W, 25°S-5°N), as these coincide with the centre of the field of view and hence optimum GLM detection.^43–45^

### 2.2 Deriving lightning NO_*x*_ production rates from LIS radiances

We adopt the approach Wu *et al.*^32^ developed to estimate LNO_*x*_ production rates over the US using satellite observations of lightning flash energies from the LIS and GLM instruments. We first calculate separate TRMM-LIS and ISS-LIS scaling factors, *β*, using eqn (1). *β* corrects for the very small fraction of total lightning flash energy detected by the LIS instrument, is in units of (sr m^−2^ µm)^−1^, and is distinct for the two LIS sensors, thus also accounting for systematic differences in lightning flash energies between the two LIS sensors.1
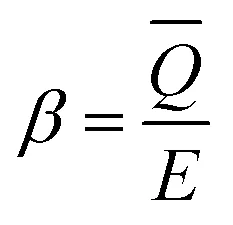
*Q̄* is the global, multiyear mean lightning flash radiance energy observed by either the TRMM-LIS or ISS-LIS, in J sr^−1^ m^−2^ µm^−1^. *E*, the total energy, in J fl^−1^ is calculated using eqn (2).2
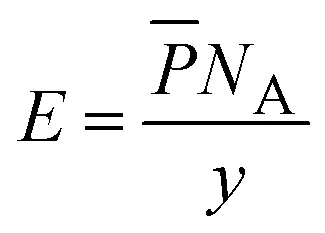


In eqn (2), *y* is the thermochemical NO_*x*_ yield, *P̄* is the global mean LNO_*x*_ production rate, and *N*_A_ is Avogadro's number. We use *y* = 9 × 10^16^ molecules J^−1^ from Navarro-González *et al.*^46^ and *P̄* = 265 mol fl^−1^ calculated by Allen *et al.*^37^ using the Earth Networks Total Lightning Network (ENTLN). Our *y* value is within range of others reported in the literature, such as 4.5–8.6 × 10^16^ molecules J^−1^,^47^ 1 × 10^17^ molecules J^−1^,^48^ and 9 ± 2 × 10^16^ molecules J^−1^.^27^ 265 mol fl^−1^ is similar to the global estimate of 280 mol fl^−1^ obtained using the GEOS-Chem model and upper tropospheric NO_2_ mixing ratios from cloud-slicing Ozone Monitoring Instrument (OMI) total NO_2_ columns.^23^ After *β* is calculated, LNO_*x*_ production rates, *P*, in mol fl^−1^, are calculated using eqn (3) for each 0.5° × 0.625° hourly gridsquare.3
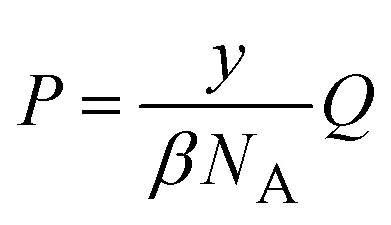


*Q* are the gridded 0.5° × 0.625° LIS lightning flash radiances for each sensor in J sr^−1^ m^−2^ µm^−1^ from Section 2.1. As *β* is calculated using global mean lightning radiances, positive biases from using mean rather median *Q* values (Section 2.1) are minimized.

The resultant 24-hour mean LNO_*x*_ production rates range widely from 2 to 30 000 mol fl^−1^. This range far exceeds the 30–700 mol fl^−1^ from the Schumann and Huntrieser review^6^ and will lead to anomalously large LNO_*x*_ emissions in CTMs, affecting model representation of NO_*x*_, O_3_ and NO_*x*_ reservoir compounds. To prevent this, we scale extreme LNO_*x*_ production rates to be within 30–700 mol fl^−1^. Values <30 mol fl^−1^ are set to 30 mol fl^−1^ and values >700 mol fl^−1^ are set to 700 mol fl^−1^. Extreme values that are scaled account for 5.5% of valid data for TRMM-LIS and 26.2% for ISS-LIS. The extreme values occur if data coverage is low, causing more extreme values for ISS-LIS, particularly over oceans.

TRMM and ISS annual production rates are combined to cover 48°S to 48°N by averaging production rates from both instruments for grid boxes where both exist or using the production rate from a single instrument for grid boxes where there is only a single value. Grid boxes with no data from either sensor within and beyond TRMM and ISS coverage are assigned the global mean value of *P̄*, 265 mol fl^−1^, and have no hourly variability.

### 2.3 The GEOS-Chem chemical transport model

We use the GEOS-Chem emissions processing package Harmonized EMissions COmponent (HEMCO) version 3.0.0 (https://doi.org/10.5281/zenodo.4984639) in its standalone configuration to replace the default static Murray *et al.*^12^ inventory LNO_*x*_ production rates with our updated annual LNO_*x*_ production rates (Section 2.2) and hourly gridded scaling factors. The static LNO_*x*_ production rates we replace are 500 mol fl^−1^ north of 35°N and 260 mol fl^−1^ everywhere else that was implemented so that the modelled vertical distribution of O_3_ matched that observed with ozonesondes and was also justified by longer flash channels in the extratropics than the tropics that were assumed to produce more NO_*x*_ per flash.

In implementing our production rates in HEMCO, we replace the static values with the gridded 24-hour multiyear mean production rates derived as detailed in Section 2.2. We then calculate hourly scaling factors as the ratio of hourly to 24-hour mean LIS flash radiances for direct input to HEMCO. HEMCO is on a global grid at a horizontal resolution of 2° latitude × 2.5° longitude over 47 vertical layers (30–35 in the troposphere) extending to 0.01 hPa (∼80 km). The lightning NO_*x*_ production rates are regridded from 0.5° latitude × 0.625° longitude to the HEMCO grid resolution *via* interpolation using regridding routines already in HEMCO.^49^ We use HEMCO to simulate 4 years of LNO_*x*_ emissions from December 2015 to November 2019 to quantify long-term global means and variability in LNO_*x*_ emissions.

Lightning flashes in HEMCO are first determined using the Price and Rind^9^ convective cloud top height parameterisation and scaled spatially to match OTD-LIS climatology, as detailed in Murray *et al.*^12^ LNO_*x*_ production rates are then applied to lightning flashes and the resultant 2D LNO_*x*_ emissions are distributed vertically using profiles from Ott *et al.*^21^ These profiles are derived from high resolution cloud-resolving model simulations that capture the dynamics of thunderstorm development and associated lightning activity. Separate profiles are used for tropical and midlatitude LNO_*x*_ and are normalised to local cloud top heights.

We apply the updated LNO_*x*_ production rates implemented in HEMCO to GEOS-Chem version 13.3.4 (https://doi.org/10.5281/zenodo.5764874) to determine the impact of our new gridded and hourly varying LNO_*x*_ production rates on seasonal mean NO_*x*_, O_3_, the oxidants hydroxyl (OH) and hydroperoxy (HO_2_) radicals, and the NO_*x*_ reservoir compound PAN. GEOS-Chem is at the same horizontal and vertical resolution as HEMCO. The model year sampled is December 2018 to November 2019 following a 1-year spin-up for chemical initialisation. This year is chosen, as we find the LNO_*x*_ emissions for 2019 to be most consistent with the 5-year annual mean from the HEMCO standalone simulations.

Both HEMCO and GEOS-Chem are driven with fixed, offline NASA Modern Era Retrospective analysis for Research and Applications, version 2 (MERRA-2) reanalysis meteorology that is gridded from its native 0.5° latitude × 0.625° longitude resolution to the model grids.

In GEOS-Chem, other tropospheric NO_*x*_ and volatile organic compound (VOC) sources forming O_3_ and NO_*x*_ reservoir compounds include anthropogenic emissions from the Community Emissions Data System version 2,^50^ aircraft from the Aviation Emissions Inventory Code,^51^ offline grid-independent soil NO_*x*_ emissions from Weng *et al.*,^52^ and the online Global Fire Emissions Database version 4 with small fires inventory for open burning of biomass.^53^ Biogenic VOCs are calculated online using Model of Emissions of Gases and Aerosols from Nature version 2.1.^54^ Treatment of sub-grid ship NO_*x*_ plumes is achieved with the PARAmetrization of emitted NOX (PARANOX) scheme.^55^

GEOS-Chem includes detailed gas-phase and heterogeneous chemistry, with updates from Horner *et al.*^56^ that reduce model underestimates in NO_*x*_ in the remote troposphere over oceans and throughout the global upper troposphere where lightning is a dominant source of NO_*x*_. These updates include photolysis of peroxypropionyl nitrate forming NO_2_ and photolysis of particle-phase nitrates, liberating NO_*x*_ as NO_2_ and as the reservoir compound nitrous acid that quickly photolyses to NO.

We use the GEOS-Chem output to evaluate the effect of changes in OH from perturbations in LNO_*x*_ emissions on the methane lifetime. This we determine using an inverse linear relationship between methane (CH_4_) lifetime and OH that is reasonable for small changes in OH:4
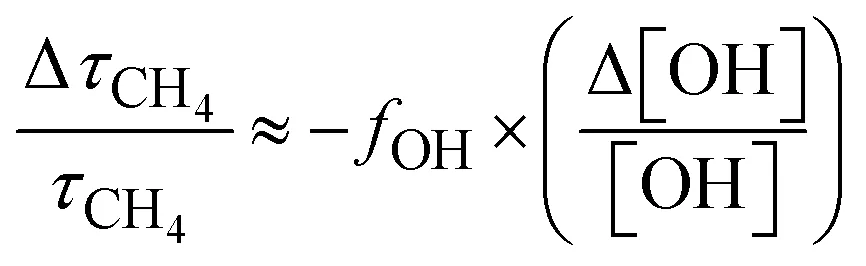

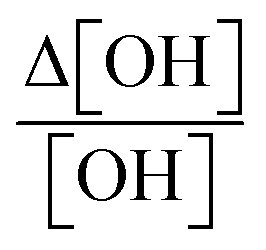
 is the relative change in global mean tropospheric OH and *f*_OH_ is 0.82, as 82% (ref. 57) of methane is lost to OH oxidation.

## 3 Results & discussion

### 3.1 LNO_*x*_ production rates derived from LIS lightning flash radiances

Fig. 1 shows maps of 24-hour mean TRMM-LIS and ISS-LIS flash radiances. Flash radiances over oceans are on average ∼0.9 J sr^−1^ m^−2^ µm^−1^ or 60% greater than over land. Land-based values range from ∼0.2 to 1.1 J sr^−1^ m^−2^ µm^−1^, with the exception of low data coverage ISS-LIS grid boxes over deserts that exceed 2 J sr^−1^ m^−2^ µm^−1^. Values over the ocean are mostly >1 J sr^−1^ m^−2^ µm^−1^. For grid boxes with at least 20 LIS observations, the TRMM averages 1.48 J sr^−1^ m^−2^ µm^−1^ over the ocean. The corresponding spatial standard deviation is 0.75 J sr^−1^ m^−2^ µm^−1^ (1.48 ± 0.75 J sr^−1^ m^−2^ µm^−1^). The average over land grid boxes is 0.81 ± 0.37 J sr^−1^ m^−2^ µm^−1^. ISS values are 0.3–0.8 J sr^−1^ m^−2^ µm^−1^ less than those of the TRMM, averaging 0.73 ± 0.37 J sr^−1^ m^−2^ µm^−1^ over the ocean and 0.47 ± 0.22 J sr^−1^ m^−2^ µm^−1^ over land. For coincident grid boxes, ISS radiances are on average 0.45 J sr^−1^ m^−2^ µm^−1^ or 31% more than those of the TRMM. The difference is greatest over the ocean at 36% more for the ISS than TRMM compared to 4.0% more over land. The two instruments are identical, so the difference results primarily from distinct viewing geometries and orbital altitudes.

**Fig. 1:**
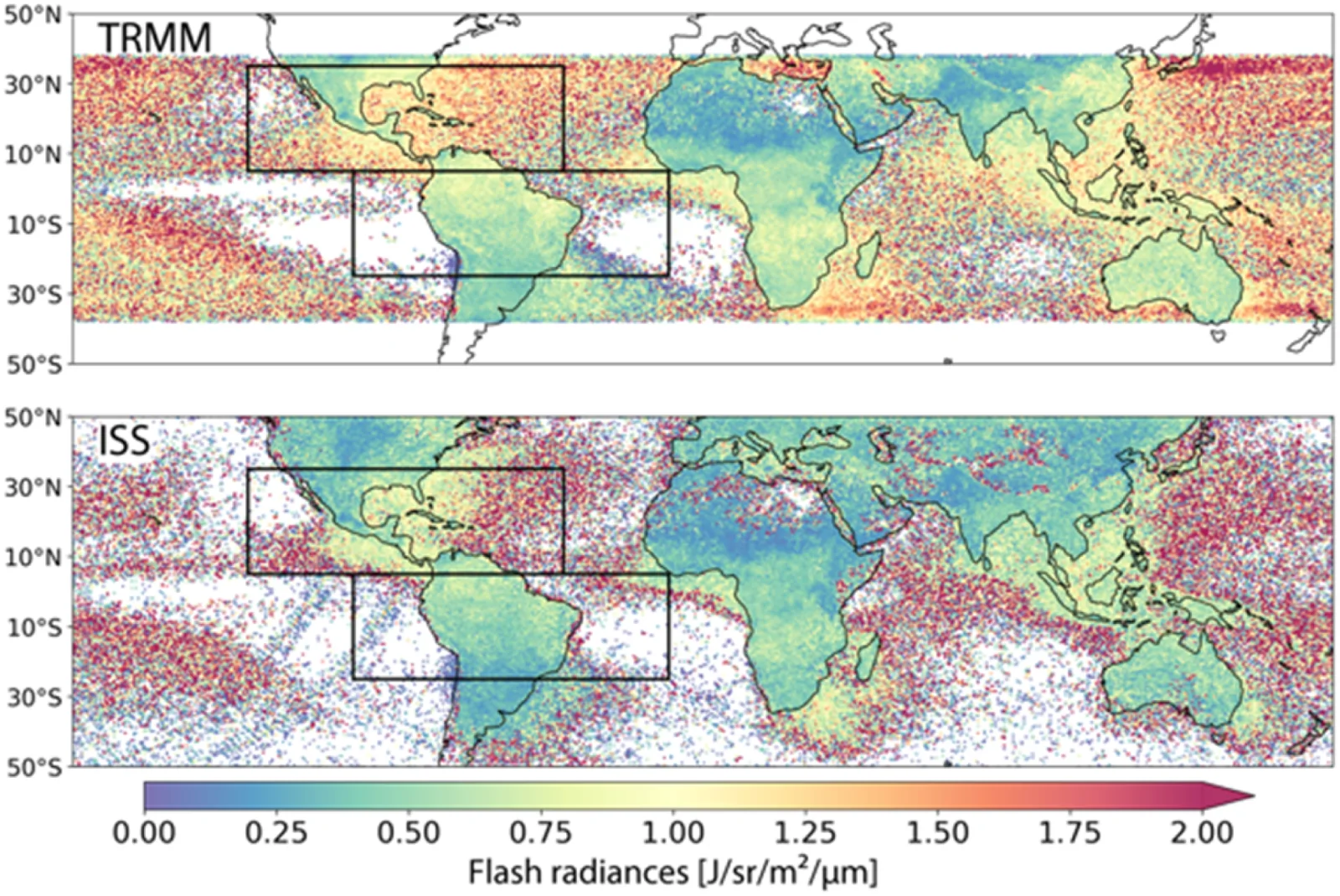
Multiyear mean 24-hour gridded LIS radiances. Panels are flash radiances from the TRMM-LIS for 2002–2014 (top) and the ISS-LIS for 2017–2022 (bottom) at 0.5° × 0.625°. Boxes show the regions sampled for comparison of the LIS and GLM hourly variability (Section 2.1, Fig. 3).

The pattern of more energetic flashes over the ocean than over land in Fig. 1 has already been reported in the literature,^40,58,59^ though the underlying reason is in dispute. Factors that have been proposed include more intense charge separation,^60^ greater charge accumulation, and larger and more mature thunderstorms over the ocean than over land.^61^ The physical attributes that have been suggested to cause these factors include differences in cloud microphysics like larger cloud droplets and fewer ice nuclei over the ocean,^62,63^ broader and more horizontally extensive anvils over the ocean,^64^ weaker and more sustained updrafts that allow charge to build up over larger regions above oceans,^65^ and higher electrical conductivity of saline ocean water.^66,67^

Fig. 2 shows hourly mean flash radiances from the two LIS instruments. Over land, both instruments have a morning peak of 0.9 J sr^−1^ m^−2^ µm^−1^ at 8 LST for the TRMM and a broader peak of ∼0.6 J sr^−1^ m^−2^ µm^−1^ at 7–10 LST for the ISS. A peak in radiances in the morning is distinct from the pronounced afternoon peak in lightning flashes.^36^ Over the ocean, TRMM hourly variability is similar to its variability over land, peaking at 8 LST and declining to a minimum at 19–20 LST. Values are systematically ∼0.3 J sr^−1^ m^−2^ µm^−1^ greater than over land. The ISS over the ocean has a broad maximum of ∼0.8 J sr^−1^ m^−2^ µm^−1^ from 6 to 12 LST declining to ∼0.6 J sr^−1^ m^−2^ µm^−1^ overnight from 17 LST to 2 LST. Sensor differences in hourly variability in radiances in Fig. 2 are due to sparse coverage of the LIS over the ocean and likely also due to differences in detection efficiencies that are not suitably corrected for with the hourly scaling factors applied to the LIS instruments (Section 2.1).^35,68^ The detection efficiency corrections reduce average biases due to high background radiances, but these are climatological means that cannot fully adjust for scene-specific detection efficiencies.

**Fig. 2:**
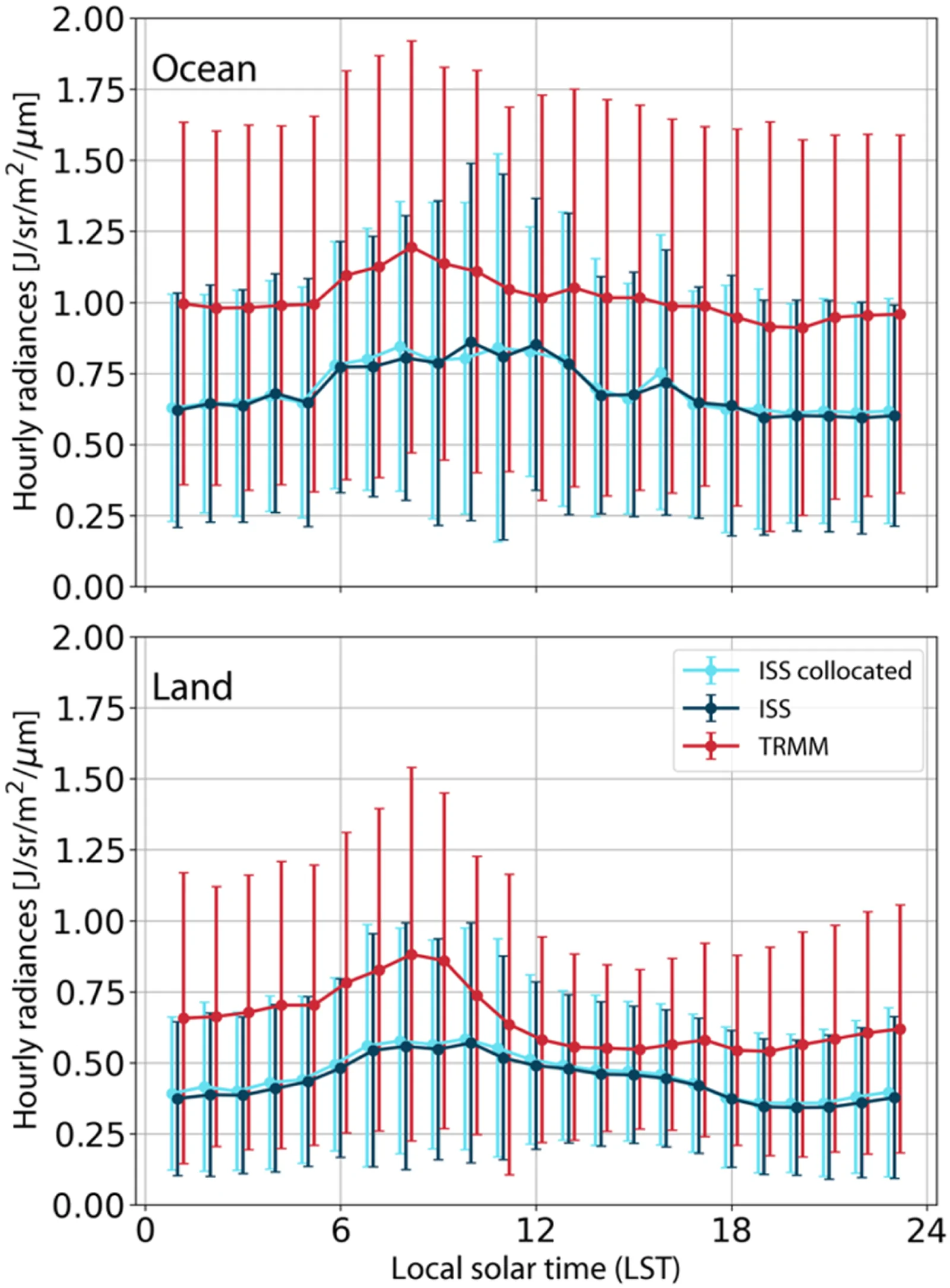
Hourly mean LIS flash radiances. Panels are hourly means over the ocean (top) and over land (bottom) for the TRMM-LIS (red), all ISS-LIS flash radiances (dark blue), and ISS-LIS flash radiances coincident with the TRMM-LIS (cyan) for grid boxes with at least 20 valid observations. Error bars are geographic standard deviations offset for visibility.

The *β* scaling factor dampens the effect of systematic differences between the sensor radiances evident in Fig. 1 and 2 on LNO_*x*_ production rates by normalising to the average retrieved radiances (Section 2.2). The *β* value for each sensor, representative of the proportion of flash energy detected, is 34% more for the TRMM (1.6 × 10^−10^ sr^−1^ m^−2^ µm^−1^) than for the ISS (1.2 × 10^−10^ sr ^−1^ m^−2^ µm^−1^). Wu *et al.*^32^ obtained a unitless *β* of 10^−22^ for GLM flash energies. The orders of magnitude difference between the GLM and LIS is because GLM energies are in 100s of fJ.

Fig. 3 compares normalised hourly variability in lightning energies for the LIS instruments and the GLM over Central America. Sampling domains are shown in Fig. 1. All three datasets exhibit an expected morning peak in global, ocean and land lightning energies typically at 8–10 LST. In the evening over land and the ocean, the GLM has a more pronounced minimum at 18–19 LST that is 30–40% less than the mean, compared to hourly values that are almost identical to the 24-h mean for the ISS and TRMM. The ISS and TRMM minima over land occur 3–4 hours later than the GLM. GLM hourly variability over land is more pronounced than the LIS sensors. GLM variability ranges from at least 50% more than the 24-h mean at 1–13 LST to less than the 24-h mean at 16–21 LST, whereas TRMM and ISS variabilities are similar to the 24-h mean for the first 6–8 hours of the day.

**Fig. 3:**
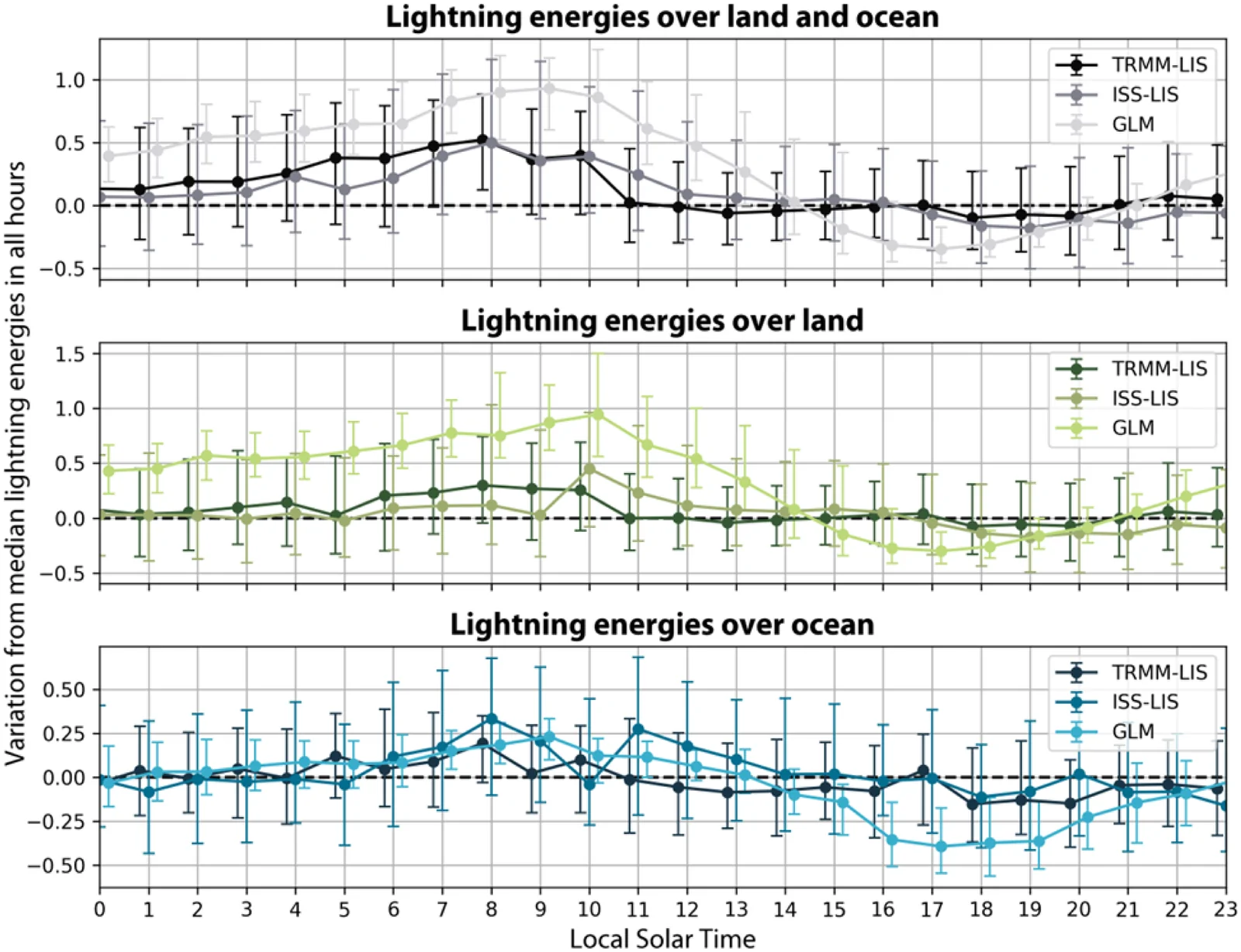
Comparison of LIS and GLM normalised hourly variation in lightning energy. Panels are normalised hourly variability [(hour minus 24-hour)/24-hour] in all coincident 0.5° × 0.625° grid boxes (top; grey), over land (middle; green) and over the ocean (bottom; blue) from the TRMM-LIS (dark shade), ISS-LIS (medium shade), and GLM (light shade). Error bars are geographic interquartile ranges offset for visibility. The coincident domains sampled are in Fig. 1.

Differences in hourly variability are likely due to a combination of observational geometry, and the timing and frequency of sampling.^69^ The latter affects the phase of storm lifecycles observed. The whole storm cycle can be monitored with the continuous coverage of the GLM. This allows for detection of peaks in lightning flash energy that may occur later or earlier in the storm evolution and that might be missed, or sampled at different intensity stages, by snapshot imagers like the LIS.^44,70^ The discrepancy that is likely most concerning for confidence in the ability to use LIS lightning radiances to model hourly variability in lightning NO_*x*_ emissions is inconsistencies in the afternoon minimum over land, as this minimum occurs soon after the prominent peak in lightning flashes over land.^71^

Fig. 4 shows the 24-hour mean LNO_*x*_ production rates. Daily mean LNO_*x*_ production rates over the oceans (309 mol fl^−1^) exceed those over land (172 mol fl^−1^) because of the larger lightning radiances over the oceans (Fig. 1). Variability in the LNO_*x*_ production rates (not shown) is determined exclusively from the variability in radiances (eqn (3)), so is similar in scale (∼60% to >100% of the hourly means) to that shown in Fig. 2. Hourly mean LNO_*x*_ production rates range from a minimum of 308.0 mol fl^−1^ at 20–21 LST to a peak of 309.5 mol fl^−1^ at 13 LST over the oceans. The range over land is greater, extending from a minimum of 167.5 mol fl^−1^ at 18 LST to a peak of 177.5 mol fl^−1^ at 8 LST. The range in land and ocean averaged hourly production rates of ∼168–310 mol fl^−1^ is similar to the range of 150–350 mol fl^−1^ in the latest review of LNO_*x*_.^8^

**Fig. 4:**
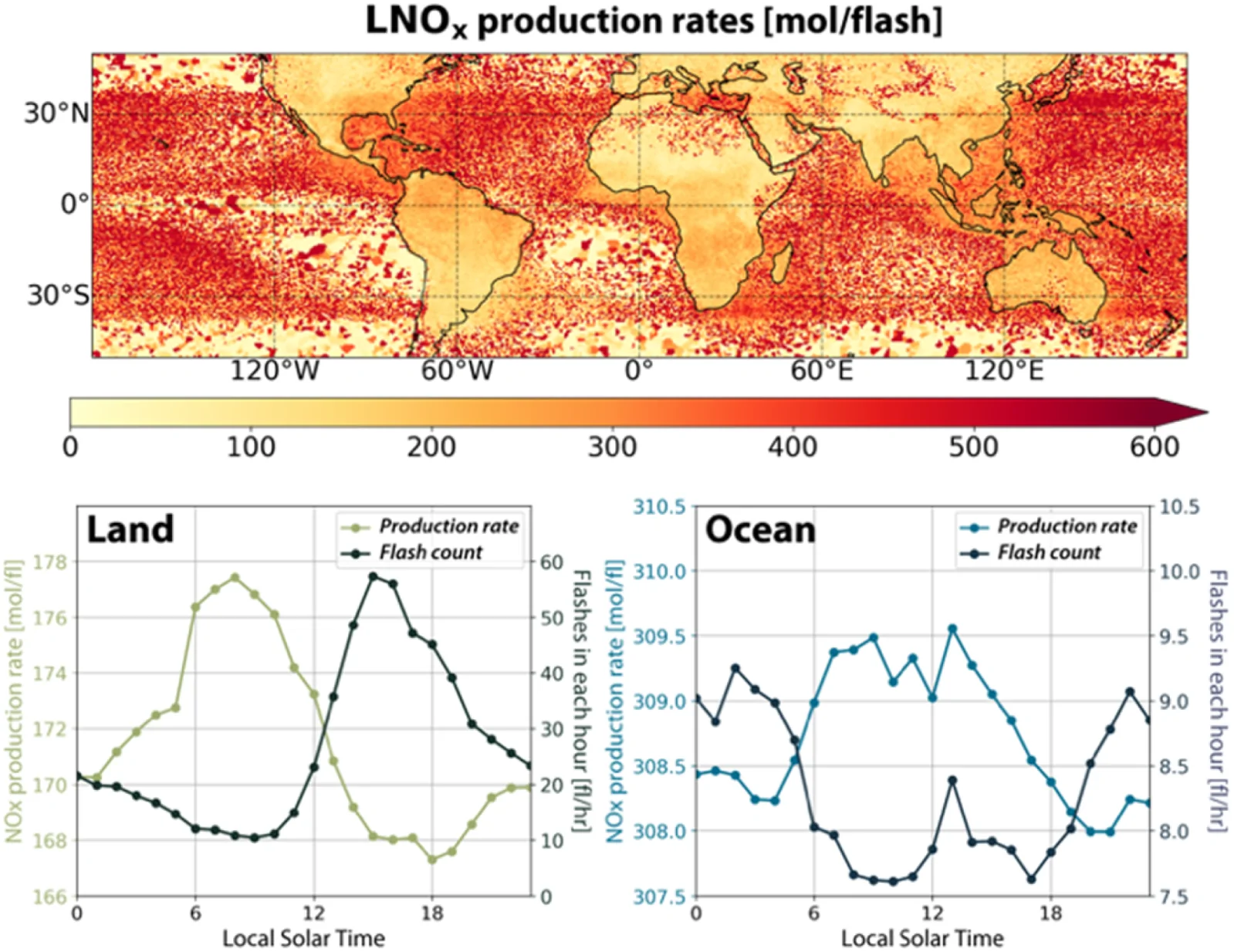
Spatial and temporal variability in LNO_*x*_ production rates. The top panel shows LNO_*x*_ production rates at 0.5° × 0.625° derived from LIS 24-hour flash radiances and the gap-filled with fixed 265 mol fl^−1^. The bottom panels are hourly mean LNO_*x*_ production rates (left axis) and the number of lightning flashes (right axis) over land (left) and the ocean (right).

The hourly variability in LNO_*x*_ production rates is opposite to that in lightning flash counts, except for the ocean peak in both LNO_*x*_ production rates and flash counts at 12–18 LST. Temporal variability in lightning flash counts is currently the only factor causing hourly variability in LNO_*x*_ emissions in CTMs. Peaks in lightning flash counts coincide with peak convection that is strongest at night and the early morning over the ocean and in the afternoon over land.^72–74^ The sole coincidence of elevated lightning flashes and radiances over the ocean at 12–15 LST is because ocean grid boxes close to coastlines are influenced by peak lightning flashes from terrestrial convection and peak lightning energies from maritime lightning.

Our use of fixed 265 mol fl^−1^ for high latitude flashes that extend beyond TRMM and ISS coverage (Section 2.1) is likely an underestimate. Ground-based lightning monitoring networks have been used to identify more powerful oceanic lightning flashes at high latitudes than anywhere else in the world,^75^ but flashes over these locations are exceedingly rare. Flashes over the Southern Ocean, for example, occur 100-times less frequently than oceanic hotspots and 1000-times less frequently than terrestrial hotspots.^75^ If our production rates are biased low, this likely has a small impact on global LNO_*x*_ emissions, as the high latitude grid boxes that are beyond TRMM and ISS coverage account for <8% of global LNO_*x*_ emissions, according to HEMCO.

### 3.2 HEMCO simulation of multiyear mean lightning NO_*x*_ emissions

The updated production rates yield global annual LNO_*x*_ emissions of 4.39 ± 0.08 Tg N year^−1^ for the 4-year simulation. This is 1.8 Tg N less than the 6.22 ± 0.23 Tg N year^−1^ obtained using the original static LNO_*x*_ production rates. The decline in LNO_*x*_ emissions is due to a lower global average NO_*x*_ yield of 265 mol fl^−1^ compared to a global weighted mean of 305 mol fl^−1^ using the original LNO_*x*_ production rates. Interannual variability in emissions, the 1*σ* standard deviation of the annual emissions, decreases from 230 Gg N to 80 Gg N with the updated LNO_*x*_ production rates. This is because of a steep decline in LNO_*x*_ emissions over northern hemisphere terrestrial regions, particularly in June–August (JJA), the timing and location of the greatest variability in each simulated year.

Fig. 5 shows the change in annual and seasonal LNO_*x*_ emissions for Dec 2018 to Nov 2019 with the updated LNO_*x*_ production rates. LNO_*x*_ emissions for these 12 months of 4.33 Tg N is ∼0.2 Tg N more than the multiyear mean. LNO_*x*_ emissions peak in JJA at 1.34 Tg N and are at a minimum in December–February (DJF) at 0.75 Tg N, due to reduced global lightning activity in these months.^57^ This seasonality in LNO_*x*_ emissions is consistent with the original approach using static production rates, but with a less pronounced peak in JJA, due to a sharp decline in JJA emissions north of 35°N (Fig. 5). This decrease is due to the change in LNO_*x*_ production rates from a static 500 mol fl^−1^ to <250 mol fl^−1^ over land (Fig. 4). Due to this change in LNO_*x*_ production rates, seasonal total LNO_*x*_ emissions decrease by 0.9 Tg N in JJA relative to the original inventory. This exceeds the decline in other seasons of 0.4 Tg N in SON and in MAM and only 0.2 Tg N in DJF.

**Fig. 5:**
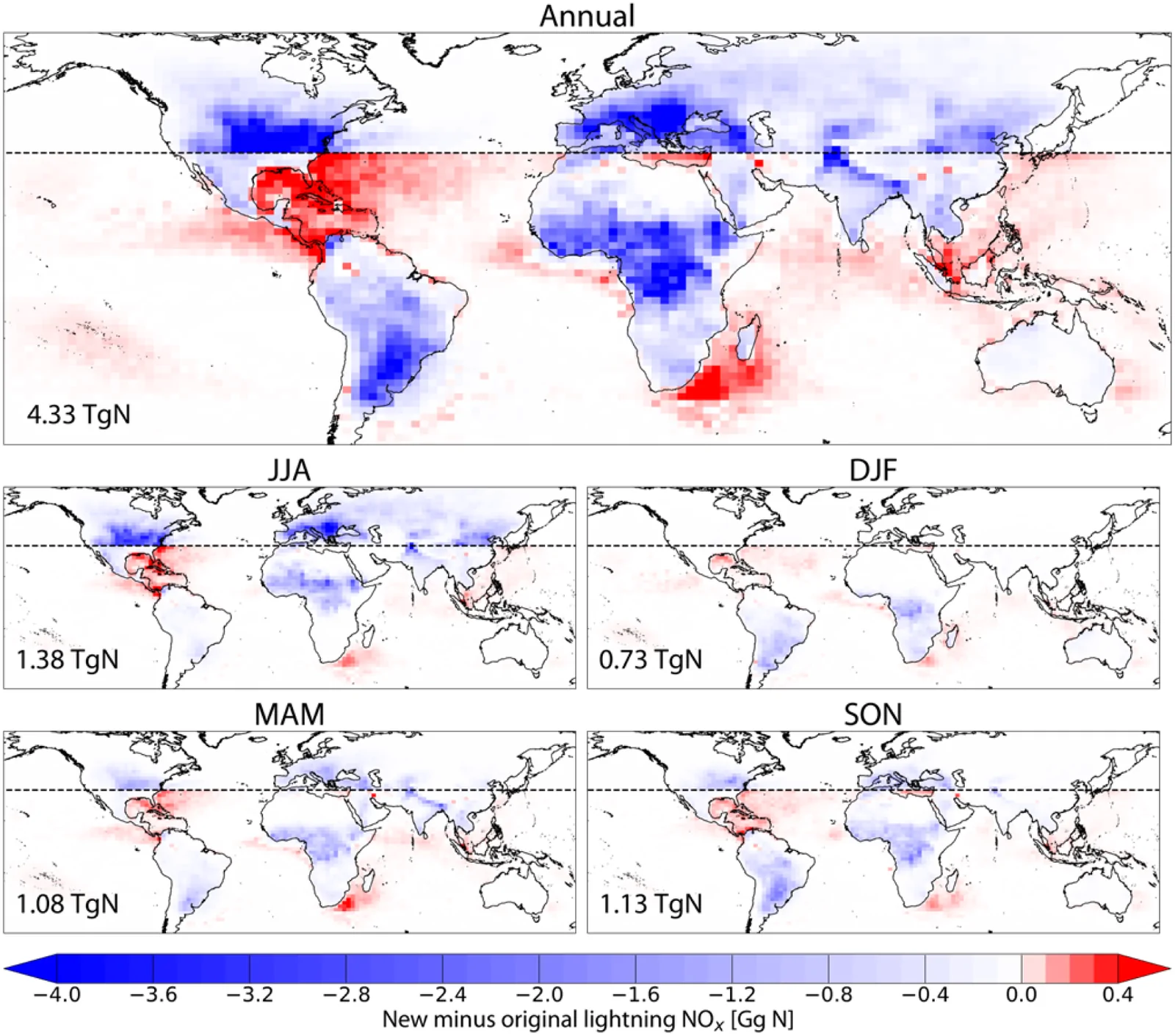
Effect of updated lightning NO_*x*_ (LNO_*x*_) production rates on LNO_*x*_ emissions. Maps are the difference in December 2018 to November 2019 LNO_*x*_ emissions (new minus original) for annual (top) and seasonal totals in June–August (middle left), December–February (middle right), March–May (bottom left), and September–November (bottom right). Dashed lines at 35°N show the boundary between the high and low static production rates in the original parameterisation (Section 2.3). Values inset are total global LNO_*x*_ emissions using the updated LNO_*x*_ production rates.

High latitude grid boxes are not shown in Fig. 5. There is negligible change in LNO_*x*_ emissions, even though northern high latitude LNO_*x*_ production rates almost halve from 500 mol fl^−1^ in the original inventory to a fixed value of 265 mol fl^−1^ in the updated inventory in the absence of observational constraints from the LIS (Section 2.2).

The updated LNO_*x*_ production rates increase the proportion of LNO_*x*_ emitted in the tropics (23°N to 23°S) from 52% of total annual emissions in the original inventory to 59% using our updated LNO_*x*_ production rates. In regions such as Central America and the Gulf of Mexico, LNO_*x*_ emissions increase by 40% in MAM (13 Gg) and JJA (26 Gg). In these tropical regions with large contribution from lightning over the ocean, the original inventory LNO_*x*_ production rate of 260 mol fl^−1^ is replaced with ∼325 mol fl^−1^ on average (Fig. 4). This is more than the 80–170 mol fl^−1^ derived by Pickering *et al.*^76^ and Allen *et al.*^77^ using NO_2_ data from the space-based instruments OMI and TROPOspheric Monitoring Instrument (TROPOMI). This discrepancy likely arises because our energy-based approach is directly sensitive to the high optical radiance of oceanic flashes, whereas the indirect column-based method may not be able to discern NO_2_ column enhancements attributable to lightning for instances with energetic lightning, but relatively few flash counts. Their method of deriving NO_*x*_ emissions is also affected by NO_*x*_ lifetime and transport errors.^7^

Compared to the original inventory, LNO_*x*_ emissions also increase by an average 0.1–0.2 Gg or 50–65% off the east coast of South Africa, an offshore lightning hotspot associated with the warm Agulhas Current, atmospheric instability from converging air masses, and frequent convective storms fuelled by moisture from the Indian Ocean.^78^ The increase in LNO_*x*_ emissions is due to greater LNO_*x*_ production rates (345 mol fl^−1^; Fig. 4) than the original 260 mol fl^−1^. The increase occurs year-round, with a slightly larger enhancement in MAM and DJF.

The spatial distribution of changes in LNO_*x*_ emissions in JJA (Fig. 5) is almost identical to those in Pérez-Invernón *et al.*^20^ They compared JJA LNO_*x*_ emissions obtained with static production rates to those obtained with spatially varying production rates calculated using an inverse relationship between lightning frequencies and LNO_*x*_ production rates. The similarity is because the locations of most energetic lightning flashes coincide with fewer flash counts and *vice versa* (Fig. 4). The only notable location where we obtain an increase and there is no evident change in Pérez-Invernón *et al.*^20^ is the hotspot off the east coast of South Africa.

The global annual 1.8 Tg N decline in emissions with the updated LNO_*x*_ production rates is due mostly to extensive decreases in LNO_*x*_ emissions year-round in tropical Africa, in South America in DJF and SON, and across North America and Eurasia in JJA and MAM (Fig. 5). These regions are either north of 35°N, where LNO_*x*_ production rates decrease from large, fixed values of 500 mol fl^−1^ for North America and Eurasia or are terrestrial lightning flash hotspots with LNO_*x*_ production rates of 125–280 mol fl^−1^ (Fig. 4) rather than the fixed 260 mol fl^−1^ in the original inventory. Examples of the latter include Central Africa and South America with intense lightning and where annual total LNO_*x*_ emissions decline by 0.6 Tg N. South America experiences peak lightning activity in DJF,^79^ causing greater absolute decline in seasonal LNO_*x*_ emissions of 0.1 Tg N (35% decrease) in DJF compared to just 0.01 Tg N (20% decrease) in JJA. In Central Africa, the spatial pattern of decline in LNO_*x*_ emissions follows the seasonal shifts in the Intertropical Convergence Zone (ITCZ) and associated lightning activity.

### 3.3 Impact of updated lightning NO_*x*_ production rates on reactive nitrogen, ozone and other oxidants

Fig. 6 shows zonal changes in tropospheric and lower stratospheric NO_*x*_, O_3_, and PAN due to updated LNO_*x*_ production rates and Fig. 7 is for OH and HO_2_. We focus first on changes coincident with LNO_*x*_ emissions changes (Fig. 5). NO_*x*_, O_3_ and PAN decrease in abundance throughout almost all of the troposphere in both seasons, indicating that the decline in continental LNO_*x*_ emissions has a greater overall impact on global atmospheric composition than the increase in maritime LNO_*x*_ emissions. Decreases in Fig. 6 are particularly pronounced in the northern hemisphere in JJA, aligning with global decreases in LNO_*x*_ emissions of 1.8 Tg N annually and 0.11 Tg N in JJA (Section 3.2; Fig. 5). In JJA, NO_*x*_ decreases by up to 20% (50 pptv) in the northern hemisphere free troposphere due to large reduction in LNO_*x*_ emissions north of 35°N and in the southern tropics boundary layer due to decline in LNO_*x*_ emissions over Central Africa (Fig. 5). The relative decline in NO and NO_2_ is similar. The decrease in NO_*x*_ in DJF is of similar magnitude to JJA, but limited to a narrower latitudinal extent peaking due north of the equator.

**Fig. 6:**
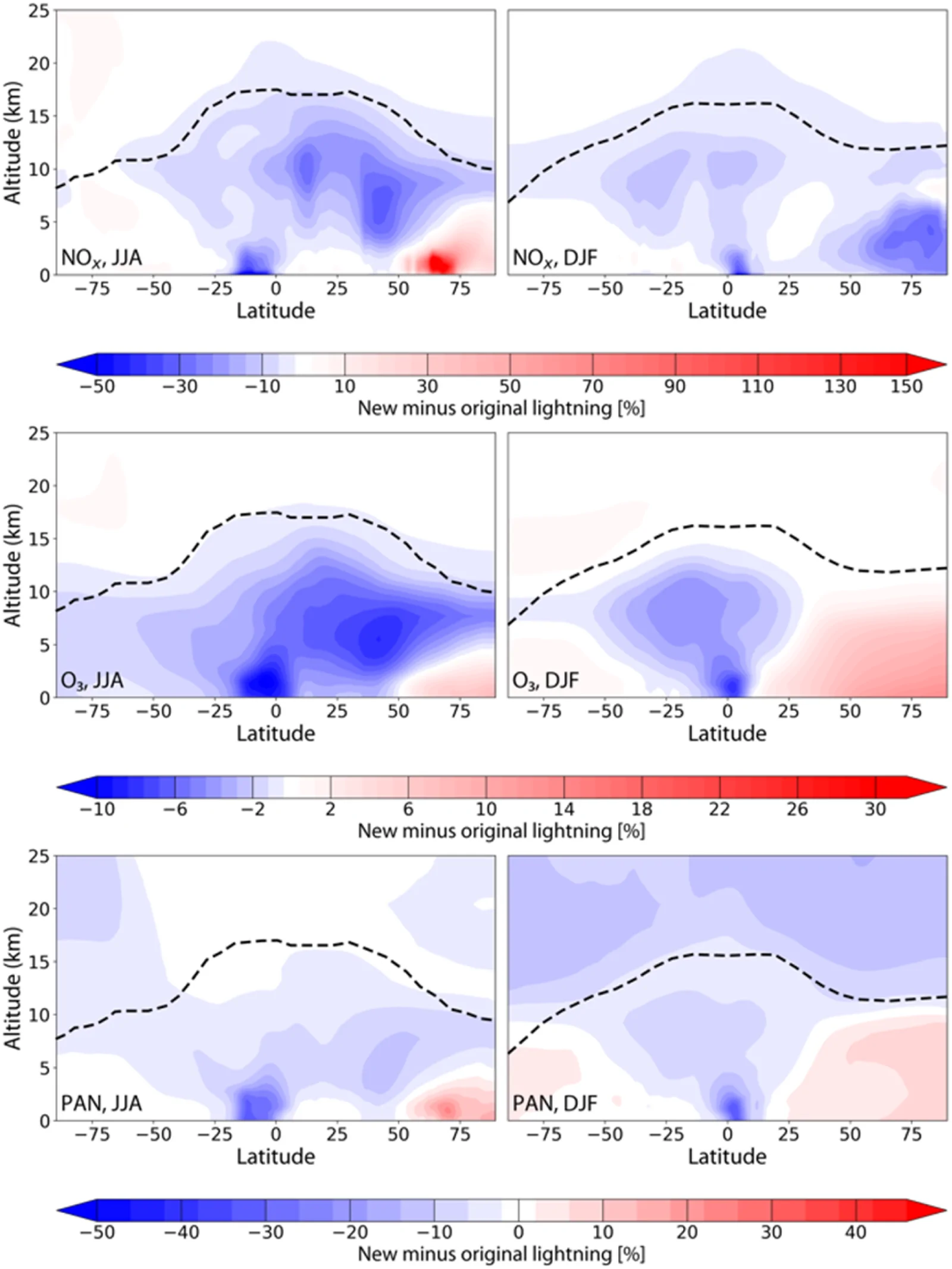
Effect of updated LNO_*x*_ production rates on atmospheric composition. Vertical profiles of the relative difference in GEOS-Chem NO_*x*_, O_3_ and PAN driven with the new and original LNO_*x*_ production rates. Columns are June–August 2019 (left) and December 2018 to February 2019 (right) and rows are NO_*x*_ (top), O_3_ (middle) and PAN (bottom). The dashed black line is the tropopause according to MERRA-2 (Section 2.3).

**Fig. 7:**
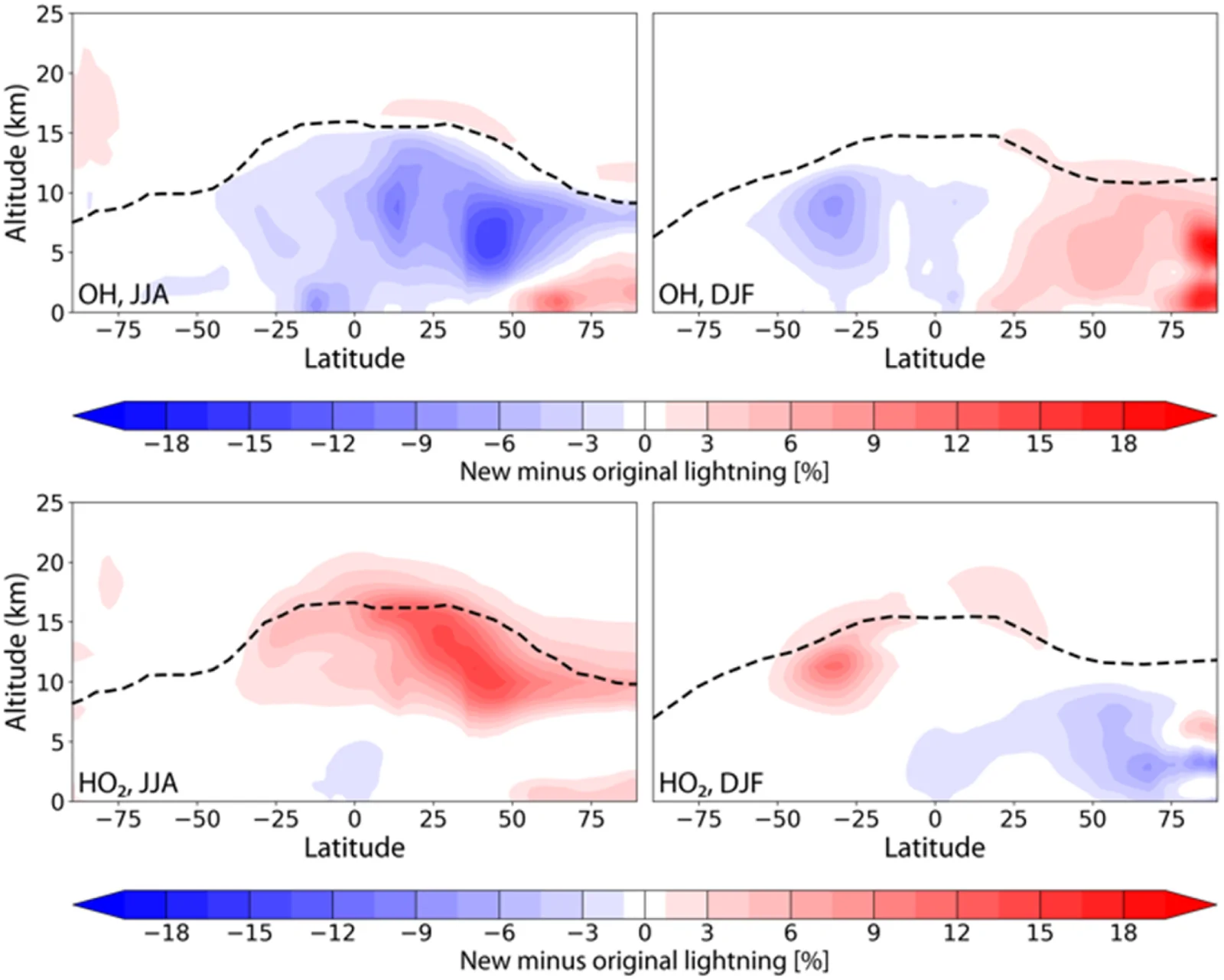
As in Fig. 6, but for OH (top row) and HO_2_ (bottom row).

The JJA and DJF declines in NO_*x*_ in the tropics and subtropics cause a corresponding decrease in tropospheric O_3_, because O_3_ production in most of the troposphere is sensitive to NO_*x*_. The decrease in O_3_ is as much as 8% (17 ppbv) in the tropical lower troposphere in JJA and DJF and in the mid-troposphere around 40°N in JJA. The decline in O_3_ at 40°N coincides with the steep decline in LNO_*x*_ production rates (Fig. 5). Decreases in PAN of 30% (40 pptv) in the tropical lower troposphere spatially correlate with the decline in NO_*x*_ in both seasons.

The changes in OH largely mirror the spatial distribution of NO_*x*_, except the winter northern middle and high latitudes. The latter results from a shift in the balance of OH and HO_2_ toward OH. The most pronounced decrease in OH of up to 10% (0.03 pptv) is in the northern hemisphere middle troposphere in JJA. In DJF, there is a small decrease in OH in the southern upper troposphere of <6%.

HO_2_ changes are overall small relative to its abundance. The largest change is an increase in the tropical and subtropical upper troposphere of up to 15% (0.4 pptv) north of the equator, as there is less NO (Fig. 6) to react with HO_2_ to form OH.

Decreases in NO_*x*_, O_3_ and OH concentrations are also documented in a recent study that updated GEOS-Chem to use a single global yield of 150 mol fl^−1^. The updated LNO_*x*_ production rate improves model agreement with integrated tropospheric NO_2_ columns from TROPOMI, O_3_ from the global ozonesonde network, and *in situ* aircraft observations of NO and OH from the NASA ATom campaign sampling the remote atmosphere in all seasons.^80^ The global 150 mol fl^−1^ value they use is derived with observations limited to North America. It falls within the broad 100–200 mol fl^−1^ range of production rates we obtain for terrestrial North America, but is less than the global terrestrial mean of 172 mol fl^−1^ and well below the global oceanic mean of 309 mol fl^−1^ (Fig. 4).

The changes in stratospheric NO_*x*_ and O_3_ in Fig. 6, restricted to the lower stratosphere, are small (<5%) relative to the stratospheric abundances of these compounds. Larger relative decline in PAN in the stratosphere is because it is not abundant above the tropopause.

The increase in abundances of all compounds in Fig. 6 and 7 in the lower troposphere in the northern high latitudes occurs where LNO_*x*_ emissions are generally absent (Fig. 5). Increases in NO_*x*_ are on average 67 pptv, equivalent to 155%. Corresponding increases in O_3_ are 4 ppbv and 7%, and in PAN are 9 pptv and 12%. PAN increases in response to increases in OH, as more VOCs are oxidized to form peroxyacetyl radicals needed to produce PAN.

We conduct a model sensitivity simulation with fixed global mean LNO_*x*_ production rates of 265 mol fl^−1^ to interpret the northern high latitude changes. The effect of a static LNO_*x*_ production rate on NO_*x*_ and OH is shown in Fig. 8. The increase in JJA NO_*x*_ and OH in the northern high latitudes shown in Fig. 6 and 7 is absent and so too is the steep decline in lower troposphere JJA NO_*x*_ in the tropics. LNO_*x*_ production rates over the continental tropics decline substantially from 260 mol fl^−1^ in the original inventory to ∼160 mol fl^−1^ in the updated inventory using spatially varying production rates (Fig. 4). A large decline in NO_*x*_ decreases the strength of terminal loss of NO_*x*_ from the reaction of NO_2_ with OH to form HNO_3_ in air transported from the tropics north,^81^ increasing the amount of NO_*x*_ subsiding as short-term reservoirs that liberate NO_*x*_ to the lower troposphere over the Arctic. Greater abundance of NO_*x*_ promotes formation of O_3_ and OH, and more OH increases VOC oxidation products that form PAN.

**Fig. 8:**
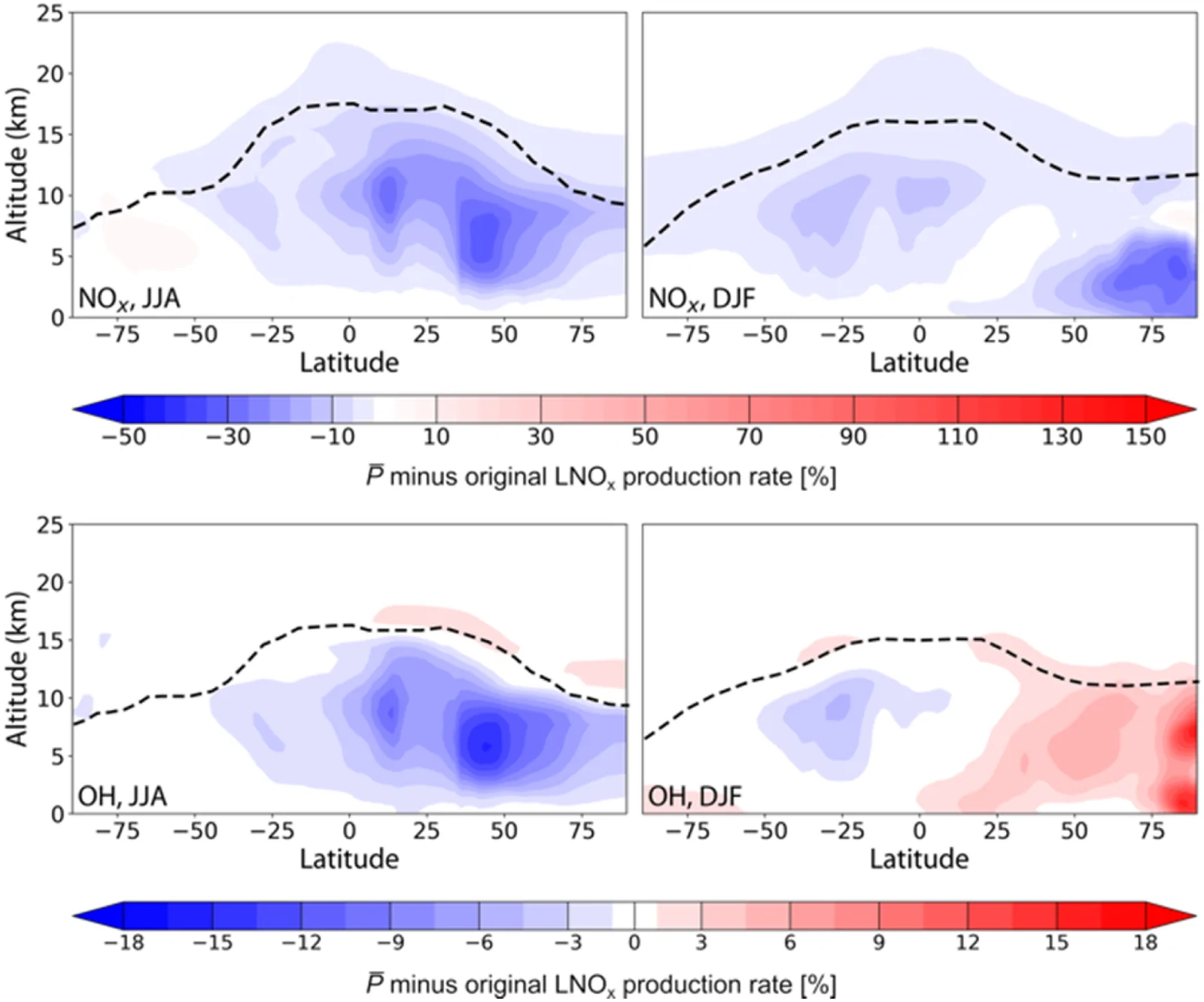
Effect of spatial variability in LNO_*x*_ production rates on NO_*x*_ and OH. Vertical profiles of the relative difference in GEOS-Chem NO_*x*_ (top) and OH (bottom) driven with the static global mean *P̄* of 265 mol fl^−1^ (eqn (2)) *versus* the original inventory. Dashed black lines denote the tropopause, as in Fig. 6 and 7.

The decline in tropospheric O_3_ resulting from the use of satellite-informed LNO_*x*_ production rates help reduce well known high biases in tropospheric O_3_ in CTMs.^82^ The average global decline in OH in the free troposphere also alters the lifetime and abundance of methane, affecting estimates of its global warming impact and emissions calculated using satellite observations of methane. The standard methane lifetime in GEOS-Chem is 8.5 years compared to ∼11 years inferred from observations.^83^ We estimate a relative decline in GEOS-Chem global annual mean tropospheric OH of ∼4% using eqn (4), corresponding to a methane lifetime increase of 0.3 years, marginally reducing the model bias from 23% to 20%.

The hourly variability in lightning flash radiances represents a potential source of uncertainty in the hourly LNO_*x*_ production rates, as independent validation is not possible and evaluation is limited to comparison with the GLM over Central America only (Fig. 3). When we simulate GEOS-Chem without this hourly variability and compare it to the model with hourly variability, we find that composition changes are almost identical to Fig. 6 and 7. An exception is that the steep >10% decline in equatorial lower tropospheric O_3_ in Fig. 6 is absent when there is no hourly variability. Hourly variability in LNO_*x*_ production rates dampens the peak in LNO_*x*_ emissions coincident with peak lightning flash frequencies (Fig. 4) and photochemistry. Instead, LNO_*x*_ emissions are shifted to the early morning when radiances peak (Fig. 4) and photochemistry is increasing, but not yet at a maximum.

## 4 Conclusions

We used lightning flash radiances from the LIS instruments onboard the TRMM satellite and the ISS to derive annual 24-hour and hourly LNO_*x*_ production rates for inclusion in the GEOS-Chem chemical transport model and the standalone version of HEMCO, GEOS-Chem's emissions processing package, to assess the impact on global LNO_*x*_ emissions and atmospheric composition. The 60% more energetic lightning over the ocean produces substantially more NO_*x*_ per flash (∼309 mol fl^−1^) than over land (∼172 mol fl^−1^).

Implementing the LIS-derived LNO_*x*_ production rates into HEMCO yields global annual LNO_*x*_ emissions of 4.39 ± 0.08 Tg N, an almost 30% reduction in annual LNO_*x*_ emissions compared to the original inventory of 6.22 ± 0.23 Tg N. The decrease is primarily from decline in production rates over land from 500 mol fl^−1^ north of 35°N and 260 mol fl^−1^ everywhere else. The greatest global decline in LNO_*x*_ emissions of 0.9 Tg N is in JJA. Even though global LNO_*x*_ emissions decrease, there are regional increases in maritime LNO_*x*_ emissions, due to large LNO_*x*_ production rates over the ocean. Specifically, Central America, the Gulf of Mexico, and the southeast coast of South Africa.

As a result of an overall global decline in LNO_*x*_ emissions, free tropospheric NO_*x*_ decreases by up to 50% in the tropical boundary layer and up to 30% in the free troposphere, causing corresponding decline in tropospheric O_3_ of up to 8–10%, aiding in addressing model overestimates in tropospheric O_3_. PAN and OH also decrease with decline in NO_*x*_, affecting redistribution of NO_*x*_ and the oxidising capacity of the atmosphere.

Our provision of hourly variability in LNO_*x*_ production rates is an advancement on previous use of space-based lightning imaging sensors. The hourly variability in lightning flash energies from LIS that drives sub-daily variability in LNO_*x*_ production rates results in a steep (>10%) decline in lower tropospheric equatorial O_3_, due to decline in LNO_*x*_ emissions in the afternoon when photochemistry peaks. Hourly variability in LIS flash radiances is only broadly consistent with hourly variability from GLM flash energies. Comparison of the LIS and GLM is limited to the tropical and subtropical Americas, so hourly variability in LNO_*x*_ production rates is a potential source of uncertainty that needs to be constrained and validated with suitable experiments and observations.

## Author contributions

RPH: conceptualisation, methodology, formal analysis, investigation, data curation, writing (original draft, and review and editing), visualisation. EAM: conceptualisation, methodology, formal analysis, investigation, writing (review and editing), supervision, funding acquisition; LTM: data curation; writing (review and editing).

## Conflicts of interest

There are no conflicts to declare.

## Data Availability

Gridded (0.5° × 0.625°) 24-hour and hourly mean lightning NO_*x*_ production rates derived in this work are available from the University College London (UCL) Research Data Repository (https://doi.org/10.5522/04/31422344).
